# Heat shock proteins, thermotolerance, and insecticide resistance in mosquitoes

**DOI:** 10.3389/finsc.2024.1309941

**Published:** 2024-01-25

**Authors:** Lindsey K. Mack, Geoffrey M. Attardo

**Affiliations:** Department of Entomology and Nematology, University of California, Davis, Davis, CA, United States

**Keywords:** HSPs (heat shock proteins), mosquito, insecticide resistance, thermotolerance, heat shock protein genes

## Abstract

Mosquitoes transmit pathogens that pose a threat to millions of people globally. Unfortunately, widespread insecticide resistance makes it difficult to control these public health pests. General mechanisms of resistance, such as target site mutations or increased metabolic activity, are well established. However, many questions regarding the dynamics of these adaptations in the context of developmental and environmental conditions require additional exploration. One aspect of resistance that deserves further study is the role of heat shock proteins (HSPs) in insecticide tolerance. Studies show that mosquitoes experiencing heat stress before insecticide exposure demonstrate decreased mortality. This is similar to the observed reciprocal reduction in mortality in mosquitoes exposed to insecticide prior to heat stress. The environmental shifts associated with climate change will result in mosquitoes occupying environments with higher ambient temperatures, which could enhance existing insecticide resistance phenotypes. This physiological relationship adds a new dimension to the problem of insecticide resistance and further complicates the challenges that vector control and public health personnel face. This article reviews studies illustrating the relationship between insecticide resistance and HSPs or *hsp* genes as well as the intersection of thermotolerance and insecticide resistance. Further study of HSPs and insecticide resistance could lead to a deeper understanding of how environmental factors modulate the physiology of these important disease vectors to prepare for changing climatic conditions and the development of novel strategies to prevent vector-borne disease transmission.

## Introduction

Mosquitoes contribute to the deaths of up to 1 million people each year globally. Across the diverse 3000 species of mosquitoes, only 9.3% have been identified as vectors of various pathogens (1). These pathogens include the malaria parasite and dengue virus. While mosquito control and disease treatment have improved significantly over the last century, the previous 20 years have seen increases in the transmission of these pathogens.

According to the World Health Organization (WHO), malaria cases steadily decreased from 2000 to 2015 from 245 million to 230 million, though since 2016, cases have again risen to 245 million. Deaths decreased from 2000-2015, from 897,000 to 577,000, but rose again to 619,000 in 2022 (2). Dengue virus has also seen massive increases in cases in the last 20 years. Since 2000, dengue cases increased from 500,000 to 5.2 million in 2019. Therapeutics for dengue are lacking (3).

Much of the increase in these diseases has been attributed to habitat expansion due to climate change and widespread insecticide resistance (4). Insecticide resistance is arguably one of the most significant challenges for pest control professionals today and plagues the public health, agricultural, and urban pest control sectors (5, 6). Resistance is developed through target-site modifications, metabolic mechanisms, cuticular modification and behavioral changes, typically with multiple mechanisms occurring within the same population (7, 8).

Mosquitoes are poikilotherms, meaning they do not regulate their body temperature and are subject to the ambient temperature in their environment. For this reason, ambient temperature influences all aspects of mosquito physiology, including metabolic rate, an important consideration for the metabolism of insecticide products (reviewed in (9)). Stresses associated with exposure to high temperatures results in initiation of a suite of responses by mosquitoes to mitigate negative impacts on physiological processes, fitness, and survival (10). This is referred to as the heat shock response and is generally facilitated by a family of stress responsive proteins called heat shock proteins (HSPs). Many genes coding for proteins within this family are associated with thermal stress, including *hsp70, hsp26* and *hsp83* (10–12).

In recent years, improvements in the quality of and access to high throughput sequencing technologies has made it easier to understand gene expression responses to external stressors, such as the xenobiotic response. This response includes a suite of detoxification and stress response enzymes including cytochrome p450s (CYPs), glutathione-s-transferases (GSTs), catalases, ATP-binding cassette (ABC) transporters, and heat shock proteins (HSPs) (13). Many of the proteins involved in this response are associated with facilitating insecticide resistance, either through mutations improving their binding efficiency for these chemicals or improving the rate at which they can breakdown insecticide (7).

Most of the research in the context of insecticide resistance has focused on ABC transporters, GSTs, and CYPs, though other stress response associated factors are likely playing a role (14). One such group is the HSPs, which act as molecular chaperones aiding in proper folding of proteins or in the repair of damaged proteins (15). Insecticide exposure induces oxidative damage through the generation of free radicals, which may be something these proteins help compensate for via a variety of mechanisms. These include protein refolding and prevention of aggregation, stabilization of reactive oxygen species, facilitating degradation of damaged molecules, and prevention of apoptosis (15–18).

The induction of HSPs by insecticides and other xenobiotics as well as meteorological factors such as temperature and humidity is a particularly important phenomenon to investigate in today’s warming climate. In California, a major center for agricultural production, new agricultural and public health pests are spreading, and the state has experienced dramatic changes to the environment over the last 20 years (19, 20). Developing a holistic understanding of the overlapping physiological processes mediating the responses to the changing climate and xenobiotic exposure will be crucial to prediction and mitigation of issues with arthropod vectors of animal and plant pathogens. Awareness of these complex interactions will provide space in which to modify existing practices that exacerbate these issues and assure adequate preparation for and mitigation of the associated public health and agricultural issues.

**Figure 1:**
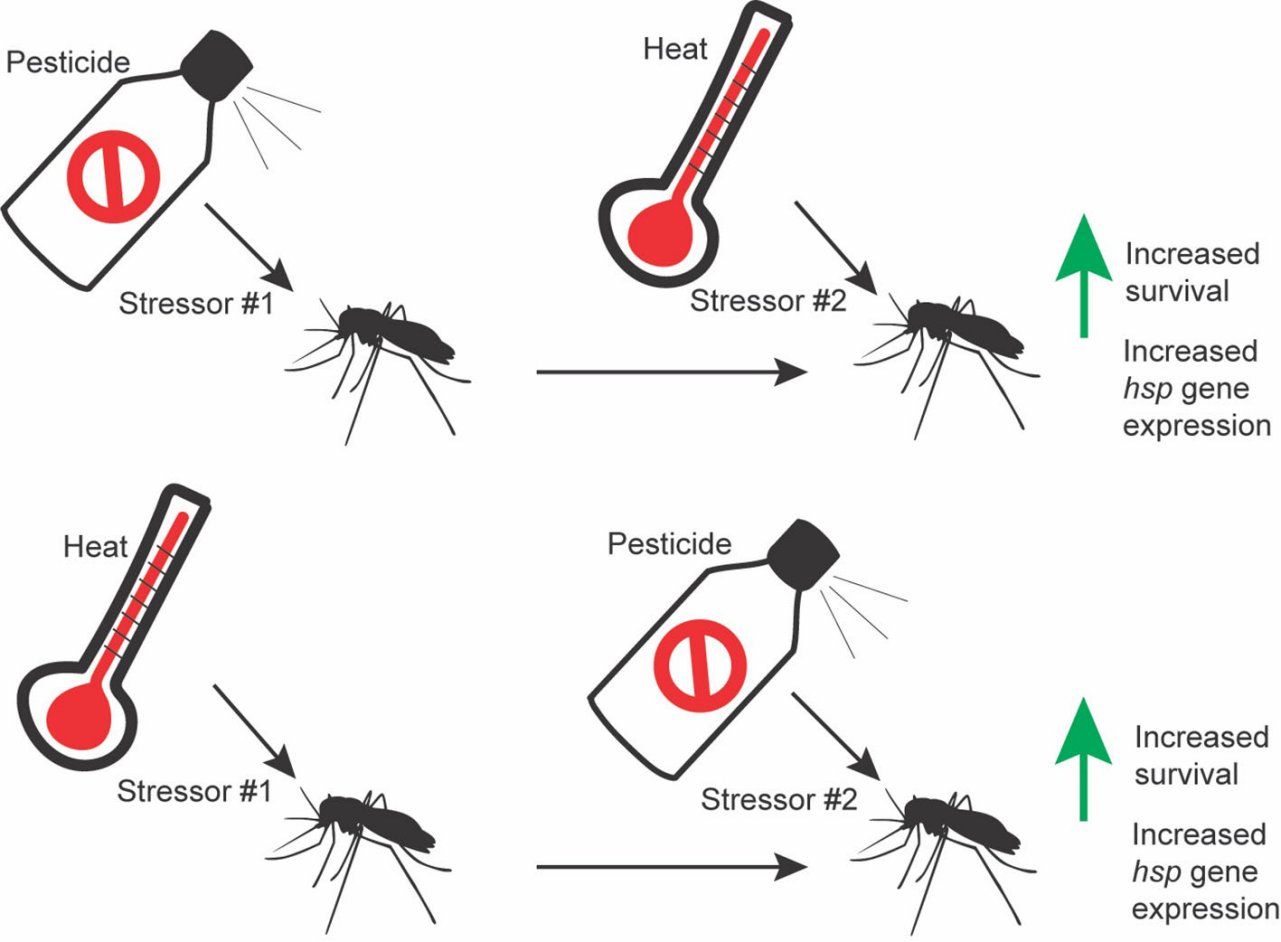
Graphical abstract representing cross tolerance between heat and insecticides.

Research demonstrates that sublethal insecticide doses induce *hsp* expression in mosquitoes and other insects, though few functional analyses have been performed (21–26). Additionally, research has shown cross-tolerance between increased temperatures and insecticides, though the direct causes of this cross-tolerance are not understood (summary in **Figure 1**) (27). Here, we review this literature and discuss future directions for research within this realm. HSPs may be an interesting target for the development of novel synergists, though further research on the physiology and direct mechanism by which these proteins are improving tolerance to insecticides is necessary.

## A brief introduction to HSPs

**Figure 2:**
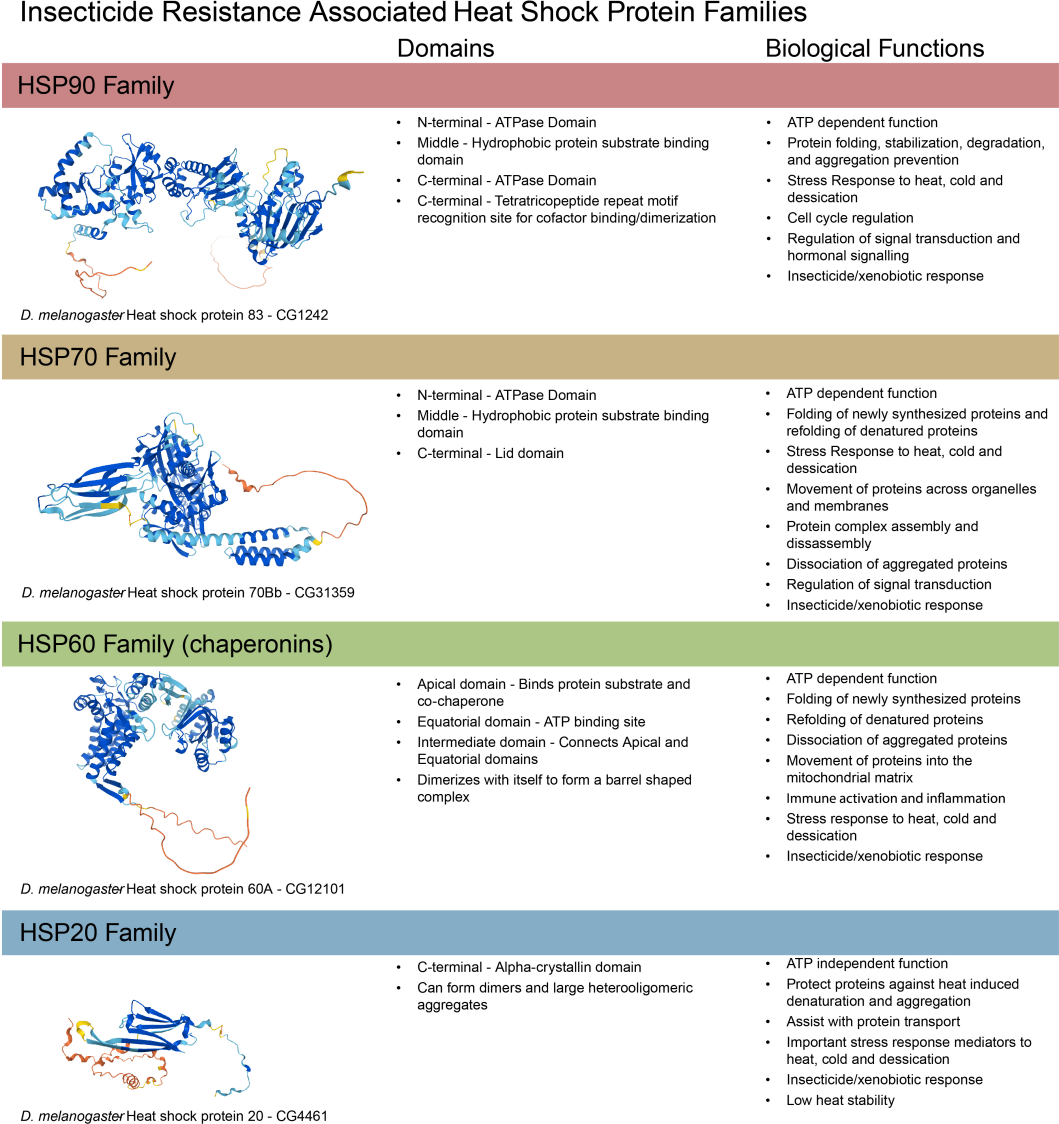
Summary of structure and function of insect heat shock protein families. Insects have have 4 families of heat shock proteins: HSP90, HSP70, HSP60, and HSP20, functionally reviewed in (15–18, 28) and summarized here. Protein structures created using Alphafold (29).

HSPs are named for their initial discovery as heat shock responsive genes. They have a wide variety of roles (reviewed thoroughly in (15)). They are well known as molecular chaperones, assisting in folding new, misfolded, or damaged proteins. They also serve important functions in cell processes like cell cycle regulation, signal transduction, and stress responses. In insects, there are 4 primary families of HSPs: small HSPs, HSP60, HSP70, and HSP90 (summarized in **Figure 2**). These are so named for their respective molecular weights in kDa. HSPs have been associated with many types of stress in insects, including temperature, hypoxia/anoxia, and oxidative stress (reviewed in (16)). In particular, their role in oxidative stress tolerance is relevant to insecticide stress and likely why they have been observed to respond to insecticides in some mosquito strains. HSPs are thought to function by stabilizing and assisting with the folding or degradation of proteins damaged by oxidative stress (reviewed in (18). Briefly, members the HSP family of proteins function through nucleotide exchange or other substrate binding measures via ATP hydrolysis in concert with various cochaperones. The commonality of oxidative stress with both insecticide exposure and heat stress may explain the cross tolerance observed in heat and insecticide resistant insects.

## Heat shock protein gene induction by insecticide

Multiple high-throughput studies of gene expression in mosquitoes have revealed increased expression of genes coding for HSPs following exposure to insecticide or in comparative studies of resistant and susceptible strains (22, 23, 30–33). The results are strain specific across studies and can vary both in the responsive genes and the dynamics of the response. A recent study in *Aedes aegypti* from California found that *hsp60A* contained a significant number of single nucleotide polymorphisms in comparison to other genes (34). This is particularly interesting, as this species of mosquito did not establish populations in California until 2013 (20). Throughout the state, mosquitoes are highly resistant to pyrethroids (35–37), and the overall variation observed in this *hsp* may be indicative of adaptation to hot, dry California weather and/or insecticide use, though further investigation is necessary.

A high throughput gene expression study examining differences between resistant field populations and a susceptible lab strain of *Anopheles sinensis*, found several *hsps* differentially expressed in the resistant groups (23). Among the 3 resistant populations tested, different *hsps* had significant changes. Interestingly, *hsp70* was downregulated in 2 populations, while *hsp90* was upregulated in all 3. The downregulation of *hsp70* may be indicative of its role as a negative regulator of the heat shock factor transcription factor (38). However, this gene displays downregulation in populations of *Drosophila* that previously experienced heat shock or a maintained heat stress, which may indicate a fitness cost of sustained expression of this gene (39). Alternatively, in a study of *Culex pipiens* examining gene expression profiles of lab selected resistant and susceptible strains, 3 *hsp70* genes and 1 *hsp83* gene were upregulated while *hsp67* was downregulated (40). No further interpretation was given to these results by the authors. Differences between the results of these two studies may be indicative of species specific, or strain specific responses.

In an RNA-seq study of resistant and susceptible strains of *Anopheles gambiae*, researchers discovered increases of *hsp* expression in mosquitoes they deemed resistant (30). Here, they used the WHO bioassay and divided mosquitoes into susceptible and resistant groups based on survival during the assay: susceptible being those that knocked down early and resistant as those who survived the 24-hour rest period. *Hsps* with increased expression included 2 *hsp20s*, 4 *hsp70s*, and 3 *hsp90s*, along with *DnaJ*. Authors attributed accumulation of these genes to an overall stress response experienced by mosquitoes after exposure to pyrethroids. Another comparative transcriptome study utilizing the WHO resistance assay for partitioning resistance groups in *Aedes albopictus* identified *hsps* that were differentially expressed in their data, but were not mentioned by gene ID, or functionally discussed (32).

An RNA-seq study characterizing the temporal genetic response of pyrethroid resistant *Ae. aegypti* from California to permethrin exposure found that 8 of the 20 genes with the largest fold change across 24 hours after exposure were *hsps* (21). These included 4 *hsp70s* and 4 *hsp20s (alpha-crystallin* and *lethal (2)-essential-for-life).* The expression of these genes also increased in the handling controls as well as the permethrin treated mosquitoes, however, expression remained elevated through 24 hours after exposure while the controls returned to baseline by 24 hours. This suggests these genes are responsive to general stressors as well. However, in *Anopheles stephensi* larvae, 7 *hsps* were shown to be downregulated across a 48-hour time period after deltamethrin exposure, which may indicate that results are strain, species, or life stage dependent (41). A study using microarrays to observe changes in gene expression over time found upregulation of *DnaJ*, 2 *alpha-crystallin A chain*, and 1 *hsp70* (42). Unrelated resistant and susceptible strains were used to assess fold changes in resistant versus susceptible mosquitoes. Again, researchers attributed these changes to general stress response induction due to permethrin exposure.

In larval *Ae. aegypti*, investigators identified 9 *hsp20* genes and 3 *hsp70* genes which increased in transcript abundance after treatment with a eucalyptus derivative (31). Interestingly, the 9 *hsp20* genes were clustered on chromosome 2, suggesting these genes are co-regulated. Their results were consistent with those found in Ingham et al., 2018, a comparative analysis of insecticide resistance and the associated transcriptional response in several *Anopheles* species, where 4 *hsp20* genes located on the same chromosome exhibited significant upregulation in resistant strains suggesting co-regulation (22).

Ingham et al. is also one of the few studies to complete functional analyses of heat shock proteins in resistance. The 4 *alpha-crystallin* genes were silenced and mortality assessed when exposed to deltamethrin, with no difference in mortality. However, RNAi knockdown of AGAP007159, an alpha crystallin B chain that was not upregulated in the microarray assay, resulted in a significant increase in mortality.

In a study examining the 2La chromosomal inversion and its role in thermotolerance and pyrethroid resistance, researchers discovered a correlation between the heterozygote form of this inversion and increased heat and pyrethroid resistance in 1 population studied (43). Additionally, researchers investigated 9 genes associated with heat tolerance and/or insecticide resistance including 6 heat shock proteins. Three *hsps (hsp70, hsp83, and hsp90)* were highly overexpressed in heat-hardened, pyrethroid exposed, and unexposed controls compared to a susceptible lab colony, suggesting these genes are associated with heat tolerance, pyrethroid response, and pyrethroid tolerance.

While the studies discussed thus far focus on *hsp* gene expression shifts in relation to insecticide exposure or resistance, two studies have examined changes to HSP protein expression in relation to bacterially derived toxins from *Bacillus thuringiensis*. In a proteomic analysis of *Aedes aegypti* larval midguts, 1 heat shock protein, HSP90, was found downregulated after exposure to the LC50 dose of Cry11Aa toxin (33). When silenced, larvae were much more tolerant of the toxin, indicating an interesting assistive effect of this protein with the toxin. However, another study found that 2 HSP70 proteins were upregulated in larvae treated with nanoparticles to deliver the Cry4Aa toxin (44). This may reflect differences in response to the specific toxins.

Overall, the *hsp70* class of genes and their respective proteins are the most well-documented in association with the response to insecticide challenge or tolerance. Not only does *hsp70* act as a molecular chaperone, but it also plays a direct role in reducing stress induced apoptosis (45). *Hsp70* may be assisting with insecticide response in both contexts. *Hsp20* is also represented in a variety of studies, often with multiple members of this gene family responding. These small heat shock proteins are characterized as chaperones, maintaining cellular processes and homeostasis. They are also ATP-independent, which could be beneficial in an energy intensive state like insecticide response (17).

Mosquitoes have a unique relationship with heat due to their use of blood as a food source. The HSP70 protein has a protective effect and positive association with fertility in *Aedes aegypti, Culex pipiens*, and *Anopheles gambiae* (11). Upon blood feeding, the mosquito body increases in temperature quickly, resulting in increased production of HSP70 in the midgut (11). Knock down of this gene resulted in a 25% reduction in egg production, indicating the importance of this protein in fertility (11). The innate ability of mosquitoes to cope with rapid shifts in temperature may make them uniquely primed to adapt to both heat stress and insecticides.

## Cross-tolerance between heat and insecticides

Generally, cross-tolerance between heat and insecticides has been studied without the addition of molecular investigation, however these studies portray the functional implications of temperature and insecticide resistance and are important to briefly review.

The first study to observe cross-tolerance between heat and insecticides in mosquitoes was published in 1996, where the authors found that larvae that experienced a heat shock event prior to exposure to propoxur, a carbamate insecticide, experienced a 50% reduction in mortality (27). Additionally, pre-treatment with propoxur prior to heat exposure proved almost as protective. In another study, researchers exposing adult *Anopheles stephensi* mosquitoes to permethrin at varying temperatures, found that those undergoing exposure between 22-28°C experience the greatest resistance ratios compared to either 16°C or 37°C (46). This differs from the previous study, as these mosquitoes did not experience a heat shock event followed by a period of recovery, so did not experience the protective effects as observed in the first mentioned study.

Larvae experience similar improvements to tolerance when adapted to high temperatures. A study in *An. stephensi* which larvae were adapted to high temperatures and then exposed to malathion as adults found an increase in survival of 2.4 to 3.1 fold at 37°C and 0.96 to 1.1 fold increase at 39°C, so it seems that the benefits of heat adaptation may be temperature dependent (47). However, in a study examining *Culex pipiens* raised at 20°C or 24°C, larvae raised at the higher temperature demonstrated higher mortality when treated with chlorpyrifos (48). Alternatively, *Cx. pipiens* that experienced a heat shock (30°C for 48 hours) prior to exposure to chlorpyrifos had much lower mortality than those raised at a constant temperature (49). The disparities in outcomes between these studies may be due to the significant differences in experimental temperature challenges or could potentially reflect strain specific adaptations.

Reciprocally, insecticide resistant strains demonstrate tolerance of higher temperatures. Another study considering differences between thermotolerance in susceptible and resistant *Cx. quinquefaciatus* found that resistant mosquitoes were more tolerant to high temperatures than their susceptible counterparts (50). The same group found that *Cx. quinequefaciatus* resistant to deltamethrin or lambda-cyhalothrin were more resistant to their respective chemicals after a 3 hour exposure to a high temperature (51). The use of resistant populations is important for these types of studies, as it provides information on the intersection of resistance mechanisms and heat tolerance mechanisms.

## Future directions

Many gaps in knowledge remain about the connection between heat shock proteins/genes, thermotolerance, and insecticide resistance. Mounting evidence suggests that mosquitoes experience cross-tolerance between heat and insecticides, and heat shock proteins could be the reason for this. Further studies are necessary to unravel the intricacies of this multifaceted response and to improve vector control decisions in the context of rising temperatures.

Understanding the relationship between HSP gene expression and protein levels is an important first step in elucidating how these genes respond to various stimuli. Mechanistic follow-up experiments, involving either the knockdown or overexpression of specific HSPs, will be crucial to assess the role of these proteins in insecticide tolerance. Such studies should be conducted across multiple species, using a range of insecticides, and at varying temperatures. The evidence suggests that there is a strong relationship between insecticide resistance and thermotolerance. However, there is significant variance in the results of the studies reviewed here, underscoring the need for further research under comparable conditions and/or targeting specific factors to provide breadth and depth to these findings. Unraveling the mechanisms and factors contributing to the relationship between environmental stress responses and insecticide resistance is essential to predict and understand the challenges facing vector control practices in the context of a warming climate.

## Author contributions

LM: Writing – original draft, Writing – review & editing. GA: Writing – review & editing.
